# Trajectories of physical activity following digital education and exercise therapy—a longitudinal registry study among 15,197 persons with knee or hip osteoarthritis

**DOI:** 10.1186/s12891-026-10590-w

**Published:** 2026-10-11

**Authors:** Ali Kiadaliri, Armaghan Mahmoudian, Leif E. Dahlberg, Andrea Dell’Isola, Eva Ageberg, L. Stefan Lohmander

**Affiliations:** 1 Department of Clinical Sciences Lund, Orthopedics, Lund University, Lund, Sweden; 2 Division of Insurance Medicine, Department of Clinical Neuroscience, Karolinska Institute, Stockholm, Sweden; 3 Department of Movement Sciences and Health, University of West Florida, Pensacola, USA; 4 Arthro Therapeutics, Malmö, Sweden; 5 Department of Health Sciences, Sport Sciences, Lund University, Lund, Sweden

**Keywords:** Digital health, Osteoarthritis, Physical activity, Self-management, Trajectory

## Abstract

**Objective:**

This study examined trajectories of physical activity (PA) and associated factors among individuals with knee or hip osteoarthritis (OA) participating in a digital education and exercise therapy program. We additionally compared patterns of patient-reported outcomes (PROs) across the identified trajectories.

**Methods:**

A longitudinal registry-based study. Individuals aged 40 + years with a diagnosis of knee or hip OA—confirmed through referral documentation or based on the National Institute for Health and Care Excellence (NICE) guidelines and Swedish national criteria— participating in the digital program with responses to validated self-reported PA questions at enrolment and at least one follow-up (3-month intervals up to 1 year after enrolment) were included (*n* = 15,197). The recommended PA level was defined as ≥ 150 min/week of moderate to vigorous physical activity (yes/no). We used group-based trajectory modelling (GBTM) to identify distinct PA trajectories and multinomial logistic regression to explore associated factors. Changes in PROs from enrolment up to 1-year follow-up across PA trajectories were analysed using random intercept models.

**Results:**

The proportion of participants reaching the recommended PA levels rose from 51.4% (95% CI: 50.6, 52.2) at enrolment to 58.5% (57.1, 59.9) at one year. GBTM identified four distinct PA trajectories: persistently high (41.2%), persistently low (30.4%), increasing (16.5%), and declining (11.9%). Body mass index (BMI) followed by physical function were the most important factors associated with PA trajectory. All PA trajectories experienced clinically comparable improvements in PROs.

**Conclusions:**

About 60% of participants reached or maintained recommended PA levels. The results suggest possible PA promotion by targeted strategies for individuals with higher BMI and poorer function. Comparable improvements in PROs across PA trajectories may support the provision of education and exercise therapy to all individuals with OA regardless of their PA level.

**Supplementary Information:**

The online version contains supplementary material available at https://doi.org/10.1186/s12891-026-10590-w.

## Introduction

Osteoarthritis (OA) is a chronic condition with high prevalence and no cure, making effective symptoms management essential [1]. Clinical practice guidelines consistently recommend physical activity (PA) as a key component of first-line treatment for all people with OA regardless of the OA severity [2]. Regular PA reduces pain and improves physical function and health-related quality of life in individuals with OA [3, 4]. Given high rates of chronic conditions and increased cardiovascular mortality in people with OA [5, 6], the benefits of PA go beyond pain and physical function. Nonetheless, most people with OA do not receive the guideline-recommended first-line care and fail to achieve recommended levels of PA, i.e. 150 min of moderate to vigorous physical activity (MVPA) per week [7–9]. The reasons for this shortfall are multifactorial including disease-related barriers (e.g. joint pain, poor physical function, OA severity), demographic and clinical factors (e.g. female sex, older age, higher body mass index, comorbidities), psychological barriers (e.g. fear of movement, low self-efficacy and motivation, limited knowledge of the benefits of PA) and care-related barriers (e.g. limited PA advice and follow-up from health professionals) [8, 10].

To bridge this evidence-practice gap and to promote PA, education and exercise therapies, delivered face-to-face or digitally, are being implemented internationally [11, 12]. Specifically, a substantial growth in the adoption of digital education and exercise therapies, as an effective and accessible delivery format, has been documented over recent years in the OA care [13]. In addition to access and costs advantages over face-to-face alternatives, these digital treatments offer the potential to deliver tailored exercise and PA support based on an individual’s specific needs, preferences and capabilities, thereby potentially enhancing engagement in regular exercise and PA [13, 14]. A recent systematic review of digital rehabilitation applications in people with knee OA documented mixed findings on the effects of these applications on the level of PA with 5 out of 12 studies reported statistically significant improvements [15].

Several studies explored average PA trajectories among OA population and identified factors associated with these trajectories including age, sex, income, education, muscle strength, physical function, and relationship factors [16–18]. However, despite large individual variability in response to exercise therapy in the OA management [19], no previous study explored the different patterns of PA among individuals with knee or hip OA participating in digital exercise therapy over time and factors associated with these patterns. Identifying subgroups of individuals with different patterns of changes in PA and their characteristics can aid in tailoring education and exercise therapies and intervene more effectively [20]. For instance, if we can identify individuals who may follow a low or decreasing PA trajectories, we can monitor their treatment progress more closely or consider switching them to an alternative intervention. The present study aimed to (1) investigate the changes in PA levels over time; (2) identify subgroups with distinct PA trajectories, (3) determine baseline characteristics associated with PA trajectory, and (4) compare longitudinal patterns of patient-reported outcomes across PA trajectories among participants in a digital education and exercise program for knee and hip OA in Sweden.

## Methods

This is a retrospective observational longitudinal study based on registry data from consecutive participants of the Joint Academy^®^, a digital education and exercise therapy application for hip and knee OA in Sweden [21]. It was introduced in Sweden in 2016 as a digital version of the evidence-based structured first-line face-to-face self-management programme. This digital program, delivered as a smartphone application, is targeted towards education, exercise, and physical activity. In addition to lessons on OA, physical activity, and self-management, participants receive individualized exercises adjusted with their progression in the program. The program includes 2 to 3 daily exercises and 2 to 3 lessons per week followed by short quizzes on the topics. Participants receive regular supervision (on average, 2 digital visits per month) from a physiotherapist via telephone or video calls, with the option for asynchronous chat communication throughout the duration of the program. The program’s core content and basic package are delivered within 12 weeks, but the program can continue upon the participants’ willingness to participate. More details on the digital program can be found elsewhere [21, 22].

### Participants

OA diagnoses were verified by a licensed physiotherapist, either from referral documentation or, where these were unavailable, through a standardised telephone interview applying the NICE and Swedish national diagnostic criteria [23, 24]. Participants entered the programme either on referral from a health professional, typically a physiotherapist or orthopaedic surgeon, or by self-enrolment following online advertising and social media campaigns. For the present study, we included all individuals aged 40 + years who enrolled in the application between January 1st, 2019, and September 30th, 2021, and provided digital informed consent for research at enrolment (*n* = 16,811). From these, we excluded participants who did not report their PA level in any follow-up (*n* = 1486) and those who reported their PA level outside a ±4week window from the expected follow-up time (*n* = 128).

### Physical activity level

PA level was measured using the responses to two self-reported, validated categorical questions from the Swedish National Board of Health and Welfare (SNBHW): (1) “During a regular week, how much time do you spend exercising on a level that makes you short winded, for example running, fitness class, or ball games?” with 6 options (scores 1 to 6 with 1: 0 min, 2: <30 min, 3: 30–60 min, 4: 61–90 min, 5: 91–120 min, and 6: >120 min) and (2) “How much time do you spend in a typical week on daily physical activity that is not exercise, such as walking, cycling or gardening?” with 7 options (scores 1 to 7 with 1: 0 min, 2: <30 min, 3: 30–60 min, 4: 61–90 min, 5: 91–150 min, 6: 151–300 min and 7: >300 min) [25]. The first question is used to measure exercise time (vigorous level PA) while the second question is used to measure everyday PA time (moderate level PA). These categorical questions provided stronger validity and correlation with accelerometers than open-ended questions among Swedish adults [25]. A total PA score is computed by multiplying the score for exercise time by two and adding this to the score for everyday PA time, resulting in an ordinal scale ranging from 3 to 19. According to the SNBHW, a total PA score ≥ 11 represents the cut-off score for achieving the recommended level of PA (i.e. 150 min of moderate-to-vigorous PA per week, Table S1 in supplement) [26]. We used this cut-off score to classify the participants as reaching the recommended PA level (yes/no). Responses to PA questions were self-reported at enrolment and at 3-month intervals (± 4 weeks) up to 1-year following enrolment.

### Covariates

The variables self-reported by the participants at enrolment were grouped in 8 categories and were included as covariates: (1) *sociodemographic* including sex, age, place of residence (living in the three metropolitan municipalities of Stockholm, Gothenburg, or Malmö: yes/no), and educational attainment (< high school, high school, college/university degree); (2) *body mass index (BMI)*, (3) *coexisting conditions* including doctor diagnosed diabetes, lung diseases, balance troubles, rheumatoid arthritis, cardiovascular diseases and the OA site (knee or hip); (4) *pain* measured using numerical rating scale (NRS) pain (0–10, best-worst) and the pain subscales of the 12-item short forms of the Knee Injury and Osteoarthritis Outcome Score (KOOS-12) or Hip disability and Osteoarthritis Outcome Score (HOOS-12) (both 0-100, worst-best) [27]; (5) *quality of life* measured using the quality of life subscale of KOOS-12/HOOS-12 and the Swedish EQ-5D-5 L index score (-0.31 to 1, worst to best) [28]; (6) *physical function* measured using the function subscale of KOOS-12/HOOS-12 and 30-second chair stand test (30s CST); (7) *activity limitations* including walking difficulties (yes/no) and the daily activity impairment (0–10, best-worst) measured using the Work Productivity and Activity Impairment questionnaire [29]; and (8) *beliefs/perceptions about OA* including readiness to do exercise (“how ready are you to start doing exercise for your knee/hip on a daily basis?” 0–10, not at all ready–extremely ready), fear of movement (yes/no), wish for surgery (yes/no), and patient acceptable symptom state (PASS, “Considering your knee/hip function, do you feel that your current state is satisfactory? You should take all activities during your daily life, sport and recreational activities, your level of pain and other symptoms, and your knee/hip-related quality of life into account.” with yes/no responses) [30].

### Data analysis

Patient characteristics at enrolment were presented as mean ± standard deviation (SD) and numbers (proportions). To compare the baseline characteristics between included and excluded participants, we calculated standardized difference and used a threshold of 0.1 to identify meaningful difference [31]. We applied inverse probability of inclusion weighting to address any difference.

To compare the characteristics of participants reaching versus not reaching the recommended PA level at enrolment, we employed Poisson regression with robust error variance, and reported prevalence ratios [32]. We visualized the participant PA status from enrolment to one-year post-enrolment using a Sankey diagram. We used multilevel mixed-effects Poisson regression with robust error variance to estimate the proportion (95% confidence interval [CI]) of persons reaching the recommended PA level during follow-up.

To identify subgroups with distinct PA trajectories, we implemented group-based trajectory modelling (GBTM) using the “traj” plugin in Stata [33, 34]. GBTM is a data-driven approach that uses maximum likelihood to divide individuals into subgroups with similar trajectory of a variable over time. To model our binary outcome (i.e. reaching the recommended PA level), we used logistic distribution in the estimation. We used GBTM with dropout extension to account for possible violation of missing at random assumption [35]. Number of days from enrolment was used as the time variable. To identify the optimal number of subgroups, we iteratively estimated models with 2 to 5 groups and various trajectory shapes (i.e., linear, quadratic, and cubic). We selected the optimal number of groups based on a combination of Bayesian information criterion (BIC, smaller absolute values indicating better fit), average posterior probability of assignment (APPA, > 0.7 for each group), the odds of correct classification (OCC, > 5 for each group), group size (≥ 5% of participants assigned to each group), relative entropy (values closer to 1 reflect better fit) and mismatch between estimated and assigned group probabilities (close to 0 for each group) [33, 36].

After identifying the optimal number of groups, each participant was assigned to the trajectory group with the highest posterior probability of membership. We used multinomial logistic regression to explore the associations between covariates and identified PA trajectories. To evaluate the relative importance of 8 grouped covariates in predicting PA trajectories, we used dominance analysis based on the contribution of these covariates to the change in the McFadden pseudo-R^2^ from multinomial logistic regression (using the “domin” command in Stata) [37].

We used a linear random intercept model with robust standard errors to compare the changes in KOOS-12/HOOS-12, activity impairment, EQ-5D-5 L index score and 30s CST across the PA trajectories from enrolment up to one-year post-enrolment. Separate models adjusted for all covariates were estimated. The KOOS-12/HOOS-12 at enrolment was missing for 1422 (8.5%) of participants (included and excluded). We used multiple imputation by chained equations to fill these missing values (10 imputations) using patient-reported outcomes (PROs) other than KOOS-12/HOOS-12 and covariates, all measured at enrolment, as input for the imputation model. All statistical analyses were implemented in Stata v.18.

## Results

A total of 15,197 individuals with mean (SD) age 64.3 (8.8) years and 75.3% females were included in the study (Table 1). Compared to those excluded, the included participants were younger, had a higher percentage of individuals with knee OA, fewer co-existing conditions, less wish for surgery, higher motivation for exercise, better physical function and EQ-5D-5 L scores and a higher PA level. However, these meaningful differences disappeared after applying the inverse of inclusion weighting.

**Table 1:** Baseline characteristics

	Included	Excluded	Absolute standardized difference	
	*n* = 15,197	*n* = 1,614	Before weighting	After weighting
Female, n (%)	11,437 (75.3)	1167 (72.3)	0.067	0.009
Age, mean (SD)	64.3 (8.8)	65.9 (9.8)	0.171	0.045
Living in metropolitan cities, n (%)	2929 (19.3)	356 (22.1)	0.069	0.003
Education, n (%)				
Less than high school	1326 (8.7)	155 (9.6)	0.041	0.018
High school	5501 (36.2)	562 (34.8)	0.029	0.021
College/university	8370 (55.1)	897 (55.6)	0.010	0.012
Body mass index, mean (SD)	27.1 (4.7)	27.3 (5.0)	0.027	0.004
Patient-acceptable symptom state: yes, n (%)	2630 (17.3)	291 (18.0)	0.019	0.010
Diabetes, n (%)	844 (5.6)	145 (9.0)	0.132	0.006
Lung diseases, n (%)	1584 (10.4)	196 (12.1)	0.054	0.001
Balance troubles, n (%)	504 (3.3)	85 (5.3)	0.096	0.013
Rheumatoid arthritis, n (%)	686 (4.5)	107 (6.6)	0.092	0.001
Cardiovascular diseases, n (%)	1138 (7.5)	181 (11.2)	0.128	0.014
Knee as the index joint, n (%)	9086 (59.8)	865 (53.63)	0.125	0.015
Walking difficulties, n (%)	9939 (65.4)	1073 (66.5)	0.023	0.002
Fear of movement, n (%)	2303 (15.2)	231 (14.3)	0.024	0.009
Wish for surgery, n (%)	2295 (15.1)	318 (19.7)	0.123	0.018
Motivation (0–10), mean (SD)	9.3 (1.3)	8.8 (1.7)	0.311	0.003
NRS pain (0–10), mean (SD)	5.1 (1.9)	5.1 (2.1)	0.031	0.001
30s CST, mean (SD)	12.7 (4.3)	12.2 (4.5)	0.115	0.010
KOOS-12/HOOS-12 pain (0-100), mean (SD)	52.9 (16.0)	52.4 (17.3)	0.032	0.014
KOOS-12/HOOS-12 function (0-100), mean (SD)	62.4 (18.7)	61.5 (19.6)	0.047	0.009
KOOS-12/HOOS-12 QoL (0-100), mean (SD)	46.0 (16.7)	45.7 (17.9)	0.015	0.007
Activity impairment (0–10), mean (SD)	3.9 (2.4)	4.1 (2.4)	0.076	0.006
EQ-5D-5 L index (-0.31–1), mean (SD)	0.85 (0.17)	0.82 (0.20)	0.148	0.005
Total physical activity score (3–19), mean (SD)	11.2 (4.1)	10.6 (4.1)	0.153	0.002
Recommended level of physical activity, n (%)	7883 (51.9)	719 (44.6)	0.147	0.004

SD: Standard deviation, NRS: Numerical rating scale, 30s CST: 30-second chair stand test

At enrolment, 51.9% of the included participants reached the recommended PA level. The proportion of individuals reaching the recommended PA level was lower among females, older people, those residing outside metropolitan cities, those with lower educational attainment, higher BMI, unsatisfactory PASS, co-existing conditions, walking difficulties, wish for surgery, less motivation for exercise, and lower scores on patient-reported outcome measures (Table 2).

**Table 2:** Factors associated with reaching the recommended level of physical activity at enrolment

	No	Yes	Prevalence ratio (95% CI)
*N (%)*	7314 (48.1)	7883 (51.9)	–
Female, n (%)	5649 (77.2)	5788 (73.4)	0.91 (0.88, 0.94)
Age, mean (SD)	64.9 (8.7)	63.7 (8.9)	0.96 (0.95, 0.97)^a^
Living in metropolitan cities, n (%)	1349 (18.4)	1580 (20.0)	1.05 (1.01, 1.09)
Education, n (%)			
Less than high school	783 (10.7)	543 (6.9)	Ref (1.00)
High school	2921 (39.9)	2580 (32.7)	1.15 (1.07, 1.24)
College/university	3610 (49.4)	4760 (60.4)	1.40 (1.31, 1.50)
Body mass index, n (%)			
< 25	2017 (27.6)	3509 (44.5)	Ref (1.00)
25-29.9	3056 (41.8)	3153 (40.0)	0.80 (0.77, 0.82)
≥ 30	2241 (30.6)	1221 (15.5)	0.55 (0.52, 0.58)
Patient-acceptable symptom state: yes, n (%)	1220 (16.7)	1410 (17.9)	1.04 (1.00, 1.08)
Diabetes, n (%)	517 (7.1)	327 (4.2)	0.73 (0.66, 0.79)
Lung diseases, n (%)	847 (11.6)	737 (9.4)	0.88 (0.83, 0.93)
Balance troubles, n (%)	308 (4.2)	196 (2.5)	0.73 (0.65, 0.82)
Rheumatoid arthritis, n (%)	358 (4.9)	328 (4.2)	0.91 (0.84, 0.99)
Cardiovascular diseases, n (%)	600 (8.2)	538 (6.8)	0.90 (0.84, 0.96)
Knee as the index joint, n (%)	4356 (59.6)	4730 (60.0)	1.01 (0.98, 1.05)
Walking difficulties, n (%)	5183 (70.9)	4756 (60.3)	0.80 (0.78, 0.83)
Fear of movement, n (%)	1142 (15.6)	1161 (14.7)	0.97 (0.92, 1.01)
Wish for surgery, n (%)	1241 (17.0)	1054 (13.4)	0.86 (0.82, 0.90)
Motivation (0–10), mean (SD)	9.1 (1.5)	9.5 (1.1)	1.14 (1.13, 1.16)
NRS pain (0–10), mean (SD)	5.2 (1.9)	4.9 (1.9)	0.95 (0.95, 0.96)
30s CST, mean (SD)	11.9 (4.1)	13.5 (4.4)	1.04 (1.04, 1.04)
KOOS-12/HOOS-12 pain (0-100), mean (SD)	51.1 (16.1)	54.6 (15.8)	1.07 (1.05, 1.08)^b^
KOOS-12/HOOS-12 function (0-100), mean (SD)	59.6 (18.8)	65.0 (18.2)	1.08 (1.07, 1.09)^b^
KOOS-12/HOOS-12 QoL (0-100), mean (SD)	43.8 (17.0)	48.0 (16.2)	1.08 (1.07, 1.09)^b^
Activity impairment (0–10), mean (SD)	4.2 (2.3)	3.6 (2.3)	0.95 (0.94, 0.95)
EQ-5D-5 L index (-0.31–1), mean (SD)	0.82 (0.18)	0.87 (0.15)	1.10 (1.08, 1.11)^c^

SD: Standard deviation, NRS: Numerical rating scale, 30s CST: 30-second chair stand test.

^a^ Per 5 years; ^b^ per 10 units; ^c^ Per 0.1 units

Out of 5 possible responses from enrolment to one year after enrolment, about 52% of the participants provided at least four responses (Table S2 in supplement). The flow of the responses to PA questions from pre- to 1-year post-enrolment is displayed in the Sankey diagram (Fig. S1). The proportion of missing responses were generally comparable between those reaching and not reaching the recommended PA level at previous follow-up. The patterns of the responses showed that 3520 (23.2%) never reached recommended PA level, while 5099 (33.6%) always reached the recommended PA level and remaining individuals (43.3%) reported changes in their achievement to the recommended PA level during the observation period. The proportion of participants reaching the recommended PA level rose from 51.4% (95% CI: 50.6, 52.2) at enrolment to 57.1% (56.3, 57.9), 57.4% (56.5, 58.3), 57.9% (56.8, 59.0) and 58.5% (57.1, 59.9) at 3-, 6-, 9-, and 12-month follow-ups, respectively.

**Fig. 1:**
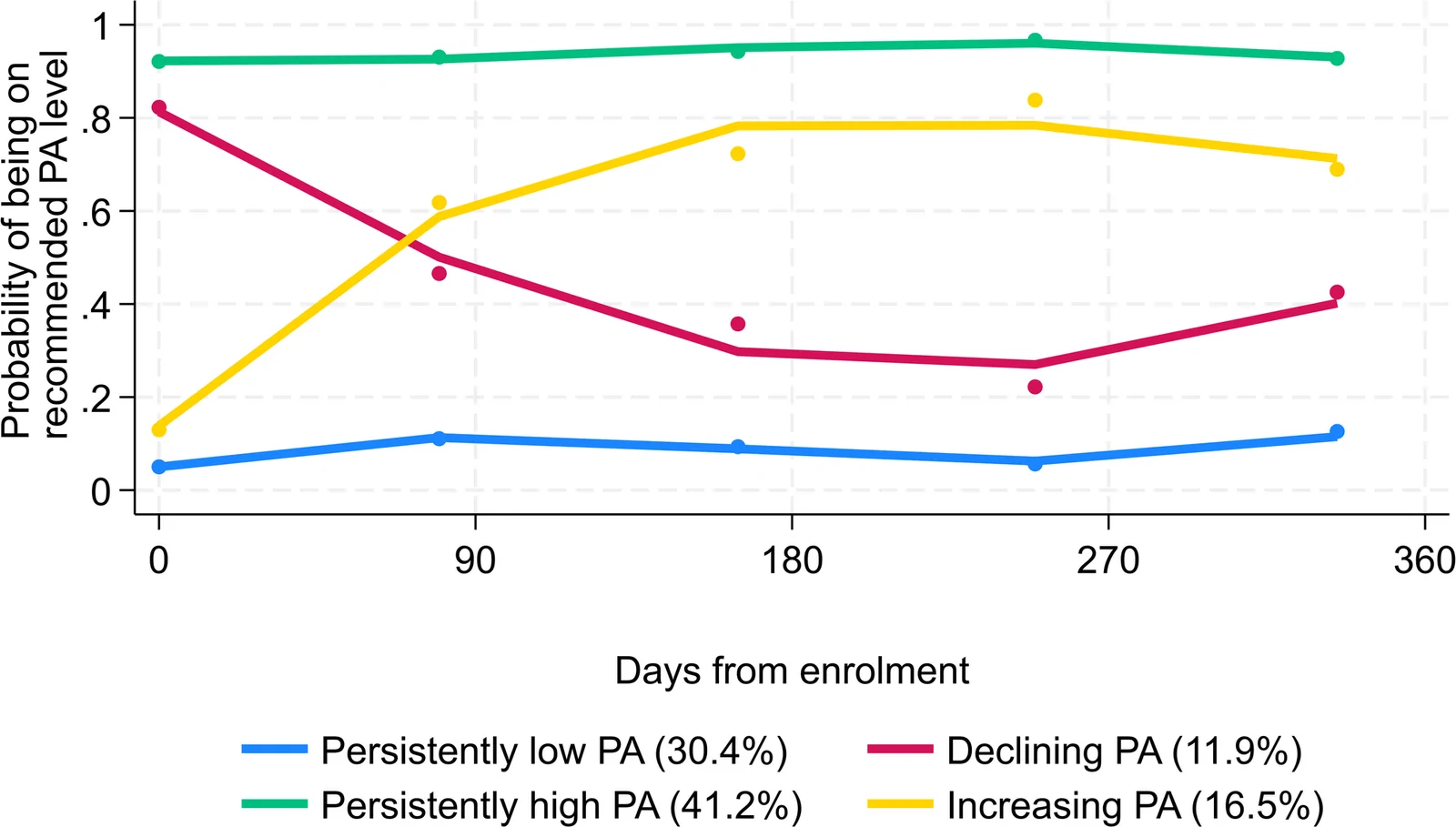
Trajectories of reaching the recommended physical activity level from enrolment up to 1-year after enrolment

In GBTM, a model with 4 groups provided the best fit to the data (Table S3 in supplement). The largest trajectory group included 41.2% of participants who persistently reached the recommended PA level (Fig. 1). This was followed by a trajectory with very low probability of reaching the recommended PA level (30.4% of participants). Two other trajectories included 16.5% and 11.9% of participant who followed an increasing and declining probability of reaching the recommended PA level, respectively. The total PA scores for these four trajectories are displayed in Fig. S2 in supplement. The mean total PA scores were about 15 and 8 in “persistently high” and “persistently low” PA levels, respectively, throughout the observation period. On the other hand, in the declining PA trajectory it decreased from about 13 at enrolment to 9–10 at follow-ups while in the increasing PA trajectory it rose from 8 to 11–12.

**Fig. 2:**
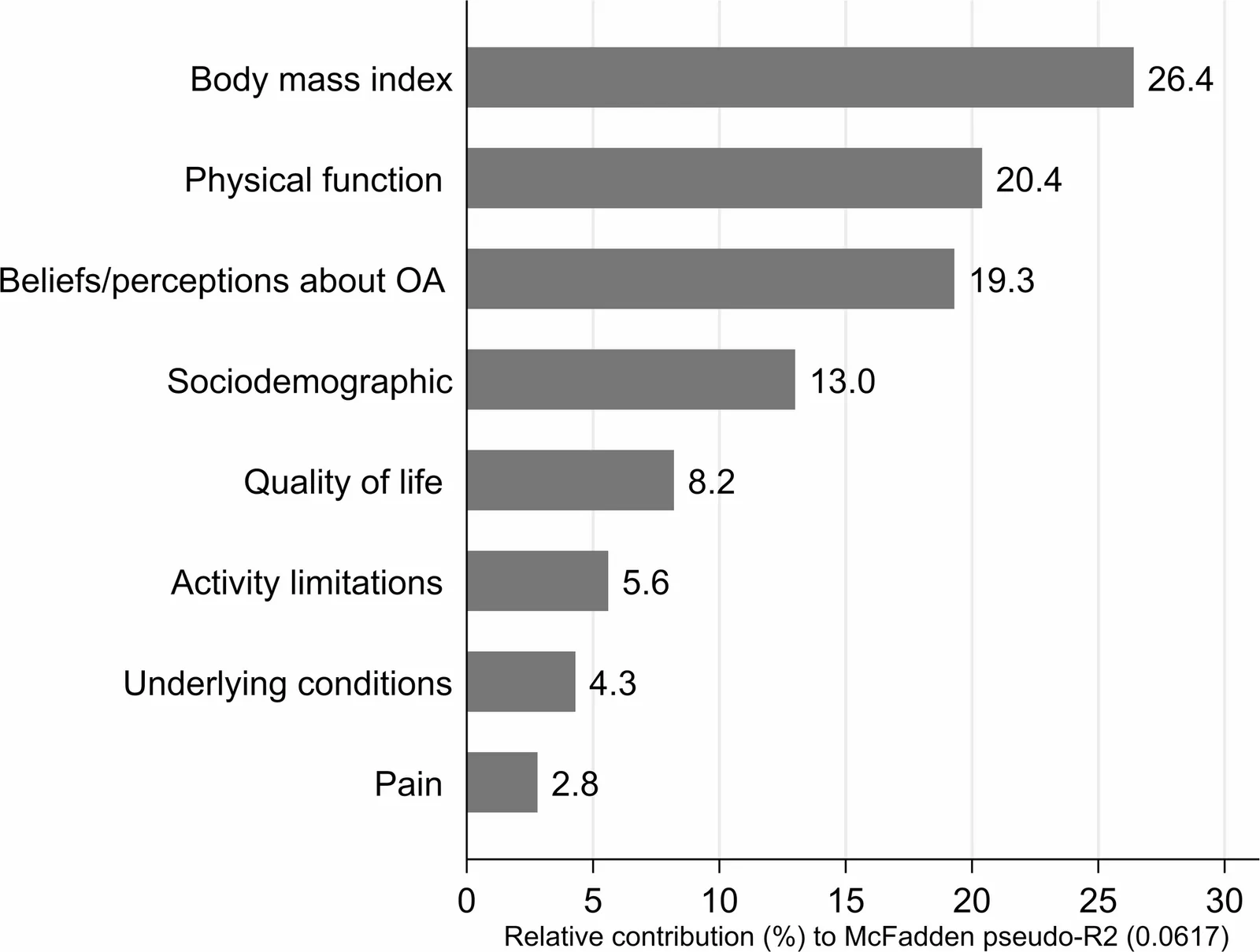
Relative contribution of baseline characteristics in predicting trajectory group membership from dominance analysis

The baseline characteristics of participants assigned to each PA trajectory are presented in Table S4 in supplement. The results of multinomial logistic regression suggested that female sex, older age, lower educational attainment, higher BMI, co-existing diabetes, lower motivation for exercise, lower physical function and EQ-5D-5 L scores were associated with higher odds of assignment to trajectory groups other than “persistently high PA” (Table 3). This model showed that all included covariates could explain 6.2% (McFadden pseudo-R^2^ =0.0617) of variations in PA trajectories and dominance analysis suggested that by accounting for 26.4% of this explained variance, BMI had the greatest relative importance in predicting PA trajectory (Fig. 2).

**Table 3:** Odds ratios (95% CI) for physical activity trajectory groups from multinomial logit model (“persistently high physical activity” group as the reference)

	DPA	IPA	PLPA
Female	1.27 (1.11, 1.44)	1.28 (1.14, 1.43)	1.38 (1.25, 1.53)
Age^a^	1.09 (1.05, 1.12)	1.11 (1.08, 1.14)	1.13 (1.10, 1.16)
Living in metropolitan cities	0.97 (0.85, 1.11)	1.00 (0.88, 1.13)	1.00 (0.90, 1.11)
Education			
Less than high school	1.00	1.00	1.00
High school	0.91 (0.74, 1.13)	0.86 (0.71, 1.03)	0.88 (0.75, 1.03)
College/university	0.76 (0.62, 0.94)	0.68 (0.57, 0.71)	0.58 (0.50, 0.67)
Body mass index			
< 25	1.00	1.00	1.00
25-29.9	1.24 (1.10, 1.40)	1.51 (1.35, 1.68)	1.71 (1.55, 1.88)
≥ 30	1.61 (1.38, 1.90)	2.08 (1.81, 2.40)	3.57 (3.17, 4.02)
Patient-acceptable symptom state: yes	1.08 (0.93, 1.25)	1.11 (0.97, 1.27)	1.10 (0.97, 1.24)
Diabetes	1.10 (0.85, 1.42)	1.27 (1.02, 1.57)	1.35 (1.12, 1.62)
Lung diseases	1.12 (0.94, 1.33)	1.03 (0.88, 1.21)	1.13 (0.99, 1.29)
Balance troubles	1.10 (0.80, 1.52)	1.12 (0.84, 1.48)	1.29 (1.02, 1.64)
Rheumatoid arthritis	1.02 (0.79, 1.32)	0.90 (0.71, 1.15)	0.93 (0.77, 1.14)
Cardiovascular diseases	0.93 (0.75, 1.15)	0.82 (0.68, 0.99)	0.96 (0.82, 1.12)
Knee as the index joint	0.93 (0.83, 1.04)	0.93 (0.84, 1.02)	0.78 (0.71, 0.85)
Walking difficulties	1.13 (0.99, 1.28)	1.08 (0.97, 1.21)	1.23 (1.11, 1.36)
Fear of movement	1.11 (0.95, 1.29)	1.07 (0.93, 1.23)	1.10 (0.98, 1.24)
Wish for surgery	1.28 (1.09, 1.51)	0.94 (0.84, 1.10)	1.03 (0.91, 1.17)
Motivation (0–10)	0.86 (0.82, 0.90)	0.84 (0.81, 0.88)	0.75 (0.72, 0.77)
NRS pain (0–10)	1.02 (0.97, 1.06)	0.99 (0.95, 1.03)	0.98 (0.95, 1.02)
30s CST	0.96 (0.95, 0.97)	0.95 (0.94, 0.96)	0.92 (0.91, 0.93)
KOOS-12/HOOS-12 pain (0-100)^b^	1.03 (0.97, 1.09)	1.04 (0.99, 1.09)	1.07 (1.03, 1.12)
KOOS-12/HOOS-12 function (0-100)^b^	0.98 (0.94, 1.03)	0.99 (0.96, 1.04)	0.98 (0.94, 1.01)
KOOS-12/HOOS-12 QoL (0-100)^b^	1.02 (0.97, 1.08)	0.96 (0.91, 1.00)	0.88 (0.84, 0.91)
Activity impairment (0–10)	1.00 (0.97, 1.03)	1.01 (0.99, 1.04)	1.02 (0.99, 1.04)
EQ-5D-5 L index (-0.31–1)^c^	0.94 (0.89, 0.98)	0.93 (0.89, 0.97)	0.91 (0.88, 0.95)

DPA: Declining physical activity; IPA: Increasing physical activity; PLPA: Persistently low physical activity.

^a^ Per 5 years; ^b^ per 10 units; ^c^ Per 0.1 units

**Fig. 3:**
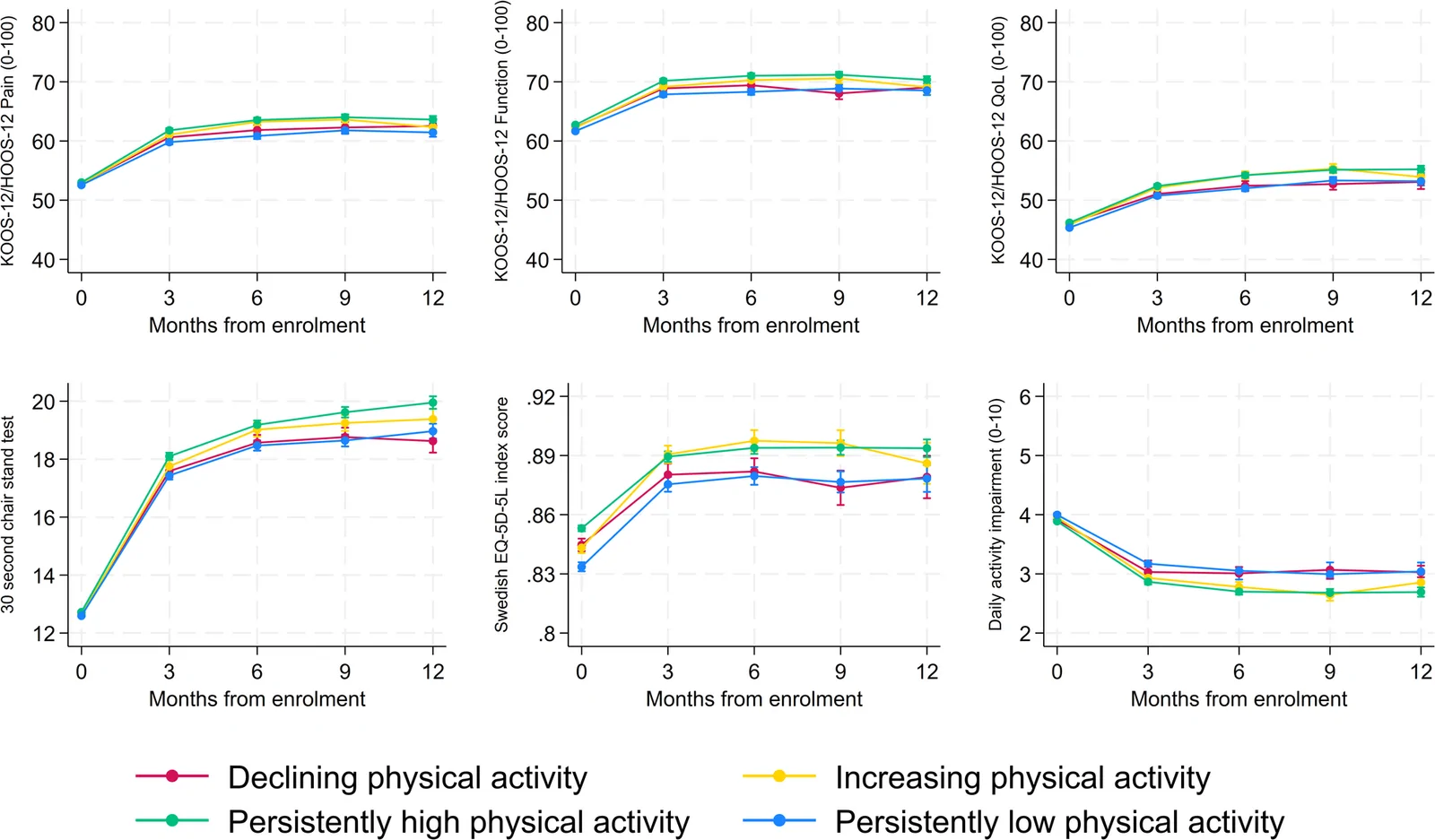
Predicted mean (95% confidence interval) in patient-reported outcomes across physical activity trajectories. Estimates obtained from random intercept model adjusted for covariates and the outcome of interest; all measured at enrolment

Comparing the longitudinal patterns of patient-reported outcomes across PA trajectories suggested that while the magnitude of improvements for “persistently high PA” and “increasing PA” groups were larger than improvements in other two groups, the differences were generally small and of uncertain clinical relevance (Fig. 3 & Table S5 in supplement).

## Discussion

In this large-scale longitudinal study, we explored PA trajectories and associated factors among over 15,000 individuals with knee and hip OA participating in a digital education and exercise program. Our findings indicated that the percentages of individuals reaching the recommended PA level rose from about 51% prior to starting the digital program to 58% at one year after participation in the program. We identified four PA trajectories over 1 year follow-up with 58% of all participants maintained or reached the recommended PA level while 42% followed a low or declining PA trajectory. Among different baseline variables, BMI was the most important factor influencing PA trajectory. All PA trajectories experienced clinically comparable improvements in patient-reported outcomes over 1-year follow-up.

The increased proportion of individuals reaching the recommended PA level in the present study aligns with evidence from the face-to-face education and exercise programs in Denmark (GLA: D^®^) [38], Sweden [39, 40], and Australia [41]. However, these studies generally reported a decrease in the proportions of physically active individuals from 3- to 12-month follow-ups. Specifically, in both studies conducted in Sweden, the percentage of individuals reaching the recommended PA level were lower at 12-months than at enrolment [39, 40]. These findings are in contrast with our findings where the percentages of individuals reaching the recommended PA level consistently improved over time. This suggests that digital delivery of education and exercise therapy with continued supervision might be an effective solution to improve and maintain exercise adherence and PA level in long term. Recent systematic reviews and meta-analyses suggested that digital interventions may improve PA levels and exercise adherence compared with face-to-face interventions among individuals with OA, even though the quality of evidence was low, with these effects apparently limited to the short term and absent in the mid- and long-term [42, 43]. Regarding the role of supervision, while the exercise sessions were performed independently rather than under real-time observation within the digital program, there were contacts with a physiotherapist (on average, two digital visits per month by telephone or video call), together with the option of asynchronous chat communication throughout the programme. Synchronous digital care, in which exercise sessions are supervised in real time, has been reported to be broadly comparable to in-person care for pain and physical function in musculoskeletal conditions, including knee and hip OA [44, 45], and provided better outcomes than asynchronous video-based exercise in axial spondyloarthritis [46]. However, whether real-time supervision of exercise sessions would further improve PA remains unexplored. It may therefore be important to explore, through head-to-head comparisons, the comparative effectiveness of digital versus face-to-face education and exercise programs on PA level among persons with OA, as well as the extent to which any benefit depends on synchronous professional supervision.

We identified four distinct PA trajectories among participants of the digital program. These trajectories reflect individual variations in adherence and responses to exercise programs reported in previous research [19, 22, 47]. The individual variations highlight the importance of tailoring interventions to individual needs for sustained behaviour change [15]. While no previous study explored variations in PA trajectory following education and exercise therapy in OA, a few studies investigated PA trajectories over 5–9 years among the general OA population mainly using data from the Osteoarthritis Initiative (OAI) [16, 17, 48, 49]. In all these studies, majority of individuals followed a low and/or declining PA trajectory, contrasting with our findings. Undergoing an education and exercise therapy and shorter follow-up in the present study compared with the OAI cohort might explain this discrepancy.

In the present study, around 42% of participants followed a persistent low (30%) or declining (12%) PA trajectory. This sizeable proportion of individuals with persistently low or declining PA may reflect the well-documented problem of low adherence to exercise recommendations in OA, especially in mid- and long-term [50]. Specifically, the smaller “declining PA” group who reached the recommended PA level at enrolment and remained close to recommended PA level (mean total PA level score 9–10 vs. the cut-off of ≥ 11) throughout follow ups, may be regarded as “almost adherent” and hence a particularly promising target for intervention. While sociodemographic and clinical characteristics of this “almost adherent” group was comparable to those with increasing PA trajectory, their wish for surgery at enrolment was closer to that in “persistently low PA” group. This may suggest that PA decline in this group is driven more by treatment expectations and waning motivation than by the physical and socioeconomic constraints characterising “persistently low PA” group, and that booster support and counselling on the continued role of PA for those wishing for surgery could help prevent relapse.

While a range of behavioural change techniques including feedback, monitoring, and tailored individualized exercises are already implemented in the digital program, this finding highlights the need for additional program modifications to promote exercise adherence and improve PA levels. Our results identified several factors associated with not reaching the recommended PA level at enrolment and following a PA trajectory other than “persistently high PA” including female sex, older age, higher BMI, lower education attainment, and lower physical function. These findings align with those reported in a recent systematic review in OA population [9] as well as findings from GLA: D^®^ Denmark and Australia [38, 41], and studies exploring PA trajectories in the OAI cohort [16, 17, 48]. Among these factors, BMI emerged as the most important factor accounting for 26.4% of explained variance in PA trajectory. BMI is a modifiable factor, and this finding suggests potential PA promotion by targeting individuals with high BMI and supports adding weight management components to the digital program.

All PA trajectories experienced clinically important improvements in pain, physical function, and health-related quality of life following participation in the digital program [51, 52]. While the magnitude of these improvements was higher for those reaching the recommended PA levels, the between-group differences were small and clinically inconclusive. This is in line with previous evidence suggesting no associations between PA level and changes in patient-reported outcomes among individuals participating in face-to-face education and exercise therapy [41, 53] or community-dwelling adults with knee OA [54]. These results reinforce the notion that any level of PA is beneficial for individuals with OA and hence education and exercise therapy should be recommended to all people with OA regardless of their PA level [4, 53].

Despite the valuable insights provided by the present study, some limitations warrant consideration. Self-reported measure of PA may introduce recall and reporting biases including social desirability bias [55]. Participants may overestimate or misclassify their activity levels, leading to potential misclassification of both the proportion reaching recommended PA and membership in the identified trajectories. Future studies could benefit from objective PA measures such as accelerometers which can also provide detailed information on activity intensity, bouts, or patterns. The lack of a control group hinders any causal inference, that is the observed improvements in PA level and patient-reported outcomes may reflect natural fluctuations in symptoms, or concurrent treatments rather than the specific effects of the digital intervention. Participation in the digital programme requires access to and comfort with digital technologies and Swedish language proficiency. It is therefore possible that our participants were more motivated, more health- and digitally literate, and potentially of higher socioeconomic position than the broader OA population, and that individuals with limited digital access or literacy, or those not speaking Swedish, are under-represented resulting in selection bias. Specifically, participants in the digital intervention are more highly educated compared with the Swedish general OA population [56] and participants of the Swedish face-to-face self-management programme [57]. Educational attainment is closely linked to digital literacy, internet access and confidence in using technology, and may therefore shape not only who enrols in a digital programme, but also who remains engaged and retained over time and who is able to translate the programme content into sustained changes in PA. Hence, the trajectories observed in the present study may partly reflect the characteristics of a socially and digitally advantaged group and not be generalisable to settings with different educational profiles. Furthermore, all participants were recruited within the Swedish health-care context, which is characterised by universal coverage and specific referral and reimbursement structures limiting the generalizability of our findings to settings with different structures. Several important factors influencing PA such as self-efficacy, symptom duration, smoking status, psychological and motivational-related factors, and country of birth were not available in the registry.

## Conclusion

We observed that the proportion of individuals reaching recommended PA level of ≥ 150 min of moderate-to-vigorous PA per week rose, on average, from 51% to 58% from prior to one year after participating in a digital education and exercise therapy for knee and hip OA. We identified four distinct PA trajectories with females, older people, those with lower educational attainment, higher BMI and poorer patient-reported outcomes at enrolment less likely to follow a high and persistent PA trajectory. Our results suggest that targeted strategies for individuals with higher BMI and poorer function may aid in promoting PA level. Comparable improvements in patient-reported outcomes across PA trajectories may support the provision of education and exercise therapy as a first-line treatment to all individuals with OA regardless of their PA level.

## Supplementary information

Below is the link to the electronic supplementary material.

Supplementary Material 1

## Data Availability

“The data that support the findings of the current study are available from Joint Academy^®^ but restrictions apply to the availability of these data, which were used under ethical permission for the current study, and so are not publicly available. Data may be made available through the corresponding author upon reasonable request and with permission of Joint Academy^®^.”

## Abbreviations

30s CST: 30-second chair stand test
APPA: average posterior probability of assignment
BIC: Bayesian information criterion
BMI: body mass index
GBTM: group-based trajectory modelling
HOOS-12: Hip disability and Osteoarthritis Outcome Score
KOOS-12: Knee Injury and Osteoarthritis Outcome Score
MVPA: moderate to vigorous physical activity
NRS: numerical rating scale
OA: osteoarthritis
OAI: the Osteoarthritis Initiative
OCC: odds of correct classification
PA: physical activity
PASS: patient acceptable symptom state
PROs: patient-reported outcomes
SD: standard deviation
SNBHW: the Swedish National Board of Health and Welfare

## References

[1] Neogi T. The epidemiology and impact of pain in osteoarthritis. Osteoarthritis Cartilage. 2013;21(9):1145–53. 10.1016/j.joca.2013.03.018 PMC3753584 23973124

[2] Conley B, Bunzli S, Bullen J, O’Brien P, Persaud J, Gunatillake T, Dowsey MM, Choong PFM, Lin I. Core Recommendations for Osteoarthritis Care: A Systematic Review of Clinical Practice Guidelines. Arthritis Care Res (Hoboken). 2023;75(9):1897–907. 10.1002/acr.25101 PMC10952362 36762545

[3] Kraus VB, Sprow K, Powell KE, Buchner D, Bloodgood B, Piercy K, George SM, Kraus WE. Physical Activity Guidelines Advisory C: Effects of Physical Activity in Knee and Hip Osteoarthritis: A Systematic Umbrella Review. Med Sci Sports Exerc. 2019;51(6):1324–39. 10.1249/MSS.0000000000001944 PMC6527143 31095089

[4] Skou ST, Pedersen BK, Abbott JH, Patterson B, Barton C. Physical Activity and Exercise Therapy Benefit More Than Just Symptoms and Impairments in People With Hip and Knee Osteoarthritis. J Orthop Sports Phys Ther. 2018;48(6):439–47. 10.2519/jospt.2018.7877 29669488

[5] Turkiewicz A, Kiadaliri AA, Englund M. Cause-specific mortality in osteoarthritis of peripheral joints. Osteoarthritis Cartilage. 2019;27(6):848–54. 10.1016/j.joca.2019.02.793 30797945

[6] Dell’Isola A, Recenti F, Englund M, Kiadaliri A. Twenty-year trajectories of morbidity in individuals with and without osteoarthritis. RMD Open. 2024;10(2):e004164. 10.1136/rmdopen-2024-004164 PMC11256023 38955511

[7] Hagen KB, Smedslund G, Osteras N, Jamtvedt G. Quality of Community-Based Osteoarthritis Care: A Systematic Review and Meta-Analysis. Arthritis Care Res (Hoboken). 2016;68(10):1443–52. 10.1002/acr.22891 26991934

[8] Wallis JA, Webster KE, Levinger P, Taylor NF. What proportion of people with hip and knee osteoarthritis meet physical activity guidelines? A systematic review and meta-analysis. Osteoarthritis Cartilage. 2013;21(11):1648–59. 10.1016/j.joca.2013.08.003 23948979

[9] Dawson ZE, Beaumont AJ, Carter SE. A Systematic Review of Physical Activity and Sedentary Behavior Patterns in an Osteoarthritic Population. J Phys Act Health. 2024;21(2):115–33. 10.1123/jpah.2023-0195 38086351

[10] Kanavaki AM, Rushton A, Efstathiou N, Alrushud A, Klocke R, Abhishek A, Duda JL. Barriers and facilitators of physical activity in knee and hip osteoarthritis: a systematic review of qualitative evidence. BMJ Open. 2017;7(12):e017042. 10.1136/bmjopen-2017-017042 PMC5770915 29282257

[11] Roos EM, Barton CJ, Davis AM, McGlasson R, Kemp JL, Crossley KM, Liu Q, Lin J, Skou ST. GLA:D to have a high-value option for patients with knee and hip arthritis across four continents: Good Life with osteoArthritis from Denmark. Br J Sports Med. 2018;52(24):1544–5. 10.1136/bjsports-2017-098904 29514823

[12] Eyles JP, Hunter DJ, Bennell KL, Dziedzic KS, Hinman RS, van der Esch M, Holden MA, Bowden JL. Joint Effort Initiative M: Priorities for the effective implementation of osteoarthritis management programs: an OARSI international consensus exercise. Osteoarthritis Cartilage. 2019;27(9):1270–9. 10.1016/j.joca.2019.05.015 31163271

[13] Johnson AJ, Palit S, Terry EL, Thompson OJ, Powell-Roach K, Dyal BW, Ansell M, Booker SQ. Managing osteoarthritis pain with smart technology: a narrative review. Rheumatol Adv Pract. 2021;5(1):rkab021. 10.1093/rap/rkab021 PMC8068316 33928214

[14] Feter N, Dos Santos TS, Caputo EL, da Silva MC. What is the role of smartphones on physical activity promotion? A systematic review and meta-analysis. Int J Public Health. 2019;64(5):679–90. 10.1007/s00038-019-01210-7 30758514

[15] Zhu D, Zhao J, Wang M, Cao B, Zhang W, Li Y, Zhang C, Han T. Rehabilitation Applications Based on Behavioral Therapy for People With Knee Osteoarthritis: Systematic Review. JMIR Mhealth Uhealth. 2024;12:e53798. 10.2196/53798 PMC11099817 38696250

[16] Wang Y, Shen Z, Xing X, Ge L, Pan F, Cai G. Association of physical activity trajectories over 8 years and risk of knee replacement: data from the osteoarthritis initiative. BMC Musculoskelet Disord. 2024;25(1):586. 10.1186/s12891-024-07710-9 PMC11282720 39061027

[17] Christensen JC, Jakiela JT, White DK. Factors Associated with Trajectories of Physical Activity Over 8 Years in Knee Osteoarthritis. Eur J Rheumatol. 2023;10(1):1–7. 10.5152/eurjrheum.2022.21155 PMC10152107 35943453

[18] Soto SH, Hales DP, Callahan LF, Rini C. Partner and Relationship Predictors of Longitudinal Physical Activity Trajectories Among Individuals with Osteoarthritis Using Latent Class Growth Analysis. Ann Behav Med. 2022;56(7):685–97. 10.1093/abm/kaab054 PMC9274998 34223870

[19] Holden MA, Hattle M, Runhaar J, Riley RD, Healey EL, Quicke J, van der Windt DA, Dziedzic K, van Middelkoop M, Burke D, et al. Moderators of the effect of therapeutic exercise for knee and hip osteoarthritis: a systematic review and individual participant data meta-analysis. Lancet Rheumatol. 2023;5(7):e386–400. 10.1016/S2665-9913(23)00122-4 38251550

[20] Frankfurt S, Frazier P, Syed M, Jung KR. Using Group-Based Trajectory and Growth Mixture Modeling to Identify Classes of Change Trajectories. Couns Psychol. 2016;44(5):622–60.

[21] Dahlberg LE, Grahn D, Dahlberg JE, Thorstensson CA. A Web-Based Platform for Patients With Osteoarthritis of the Hip and Knee: A Pilot Study. JMIR Res Protoc. 2016;5(2):e115. 10.2196/resprot.5665 PMC4912680 27261271

[22] Kiadaliri A, Horder H, Lohmander LS, Dahlberg LE. Weekly pain trajectories among people with knee or hip osteoarthritis participating in a digitally delivered first-line exercise and education treatment. Pain Med. 2024;25(4):291–9. 10.1093/pm/pnad167 38127991

[23] National Institute for Health and Care Excellence (NICE). Osteoarthritis in over 16s: diagnosis and management. In. London: National Institute for Health and Care Excellence; 2022. 36745715

[24] The National Board of Health and Welfare. National guidelines for musculoskeletal diseases – Rheumatoid arthritis, axial spondyloarthritis, psoriatic arthritis, osteoarthritis and osteoporosis – Support for governance and management. In. Sweden: The National Board of Health and Welfare; 2021.

[25] Olsson SJ, Ekblom O, Andersson E, Borjesson M, Kallings LV. Categorical answer modes provide superior validity to open answers when asking for level of physical activity: A cross-sectional study. Scand J Public Health. 2016;44(1):70–6. 10.1177/1403494815602830 26392418

[26] Welfare TSNBoHa. Validering av socialstyrelsens screeningfrågor om fysisk aktivitet. In.; 2023.

[27] Gandek B, Roos EM, Franklin PD, Ware JE Jr. Item selection for 12-item short forms of the Knee injury and Osteoarthritis Outcome Score (KOOS-12) and Hip disability and Osteoarthritis Outcome Score (HOOS-12). Osteoarthritis Cartilage. 2019;27(5):746–53. 10.1016/j.joca.2018.11.011 30593867

[28] Sun S, Chuang LH, Sahlen KG, Lindholm L, Norstrom F. Estimating a social value set for EQ-5D-5L in Sweden. Health Qual Life Outcomes. 2022;20(1):167. 10.1186/s12955-022-02083-w PMC9780618 36564844

[29] Reilly MC, Zbrozek AS, Dukes EM. The validity and reproducibility of a work productivity and activity impairment instrument. PharmacoEconomics. 1993;4(5):353–65. 10.2165/00019053-199304050-00006 10146874

[30] Ingelsrud LH, Granan LP, Terwee CB, Engebretsen L, Roos EM. Proportion of Patients Reporting Acceptable Symptoms or Treatment Failure and Their Associated KOOS Values at 6 to 24 Months After Anterior Cruciate Ligament Reconstruction: A Study From the Norwegian Knee Ligament Registry. Am J Sports Med. 2015;43(8):1902–7. 10.1177/0363546515584041 25977523

[31] Austin PC. Balance diagnostics for comparing the distribution of baseline covariates between treatment groups in propensity-score matched samples. Stat Med. 2009;28(25):3083–107. 10.1002/sim.3697 PMC3472075 19757444

[32] Barros AJ, Hirakata VN. Alternatives for logistic regression in cross-sectional studies: an empirical comparison of models that directly estimate the prevalence ratio. BMC Med Res Methodol. 2003;3:21. 10.1186/1471-2288-3-21 PMC521200 14567763

[33] Nagin DS, Jones BL, Elmer J. Recent Advances in Group-Based Trajectory Modeling for Clinical Research. Annu Rev Clin Psychol. 2024;20(1):285–305. 10.1146/annurev-clinpsy-081122-012416 38382118

[34] Jones BL, Nagin DS. A Note on a Stata Plugin for Estimating Group-based Trajectory Models. Sociol Methods Res. 2013;42(4):608–13.

[35] Haviland AM, Jones BL, Nagin DS. Group-based Trajectory Modeling Extended to Account for Nonrandom Participant Attrition. Sociol Methods Res. 2011;40(2):367–90.

[36] Klijn SL, Weijenberg MP, Lemmens P, van den Brandt PA, Lima Passos V. Introducing the fit-criteria assessment plot - A visualisation tool to assist class enumeration in group-based trajectory modelling. Stat Methods Med Res. 2017;26(5):2424–36. 10.1177/0962280215598665 26265768

[37] Luchman JN. Determining relative importance in Stata using dominance analysis: domin and domme. Stata J. 2021;21(2):510–38.

[38] Baumbach L, Gronne DT, Moller NC, Skou ST, Roos EM. Changes in physical activity and the association between pain and physical activity - a longitudinal analysis of 17,454 patients with knee or hip osteoarthritis from the GLA:D(R) registry. Osteoarthritis Cartilage. 2023;31(2):258–66. 10.1016/j.joca.2022.09.012 36272673

[39] Ernstgard A, PirouziFard M, Thorstensson CA. Health enhancing physical activity in patients with hip or knee osteoarthritis - an observational intervention study. BMC Musculoskelet Disord. 2017;18(1):42. 10.1186/s12891-017-1394-7 PMC5267429 28122519

[40] Sturesdotter Akesson K, Beckman A, Stigmar K, Sunden A, Ekvall Hansson E. Physical activity and health-related quality of life in men and women with hip and/or knee osteoarthritis before and after a supported self-management programme - a prospective observational study. Disabil Rehabil. 2022;44(16):4275–83. 10.1080/09638288.2021.1900417 33761294

[41] Bell EC, Pazzinatto MF, Wallis JA, Kemp JL, Skou ST, O’Halloran P, Crossley KM, De Oliveira Silva D, Semciw AI, Dundules K, et al. Association of baseline physical activity participation with participant characteristics and outcomes following education and exercise-therapy in people with knee osteoarthritis: A GLA:D((R)) Australia prospective cohort study. Musculoskelet Care. 2023;21(4):1470–81. 10.1002/msc.1828 37795974

[42] Baigi SFM, Kimiafar K, Ghaddaripouri K, Mehneh MR, Mousavi AS, Sarbaz M. The effect of telerehabilitation on improving the physical activity of patients with osteoarthritis: A systematic review. J Educ health promotion. 2023;12:408. 10.4103/jehp.jehp_1586_22 PMC10852179 38333155

[43] Liu L, Wang S, Ye C, Chen D, Dong H. The effects of digital health on exercise adherence and intervention outcomes in older adults with knee diseases: A systematic review. Ann Phys Rehabil Med. 2025;68(5):101952. 10.1016/j.rehab.2025.101952 40080931

[44] Corso M, Cancelliere C, Mior S, Salmi LR, Cedraschi C, Nordin M, Sci DM, Taylor-Vaisey A, Cote P. Are Nonpharmacologic Interventions Delivered Through Synchronous Telehealth as Effective and Safe as In-Person Interventions for the Management of Patients With Nonacute Musculoskeletal Conditions? A Systematic Rapid Review. Arch Phys Med Rehabil. 2022;103(1):145–54. e111. 10.1016/j.apmr.2021.09.007 34736919

[45] de Sire A, Prestifilippo E, Cofano E, Curci C, Marotta N, Russo R, Invernizzi M, Ammendolia A, Gasparini G, Familiari F. Effectiveness of real-time visual supervised telerehabilitation on functioning following knee arthroplasty compared with outpatient or home-based exercise: A systematic review of randomized controlled trials. J Experimental Orthop. 2026;13(2):e70575. 10.1002/jeo2.70575 PMC13266366 42305889

[46] Sen EN, Saritas F, Ozturk O. Synchronous and asynchronous remote core stability exercises in patients with axial spondyloarthritis: a randomized-controlled clinical trial. Ir J Med Sci. 2025;194(1):137–46. 10.1007/s11845-024-03846-8 39589722

[47] van Middelkoop M, Schiphof D, Hattle M, Simkins J, Bennell KL, Hinman RS, Allen KD, Knoop J, van Baar ME, Bossen D, et al. People with short symptom duration of knee osteoarthritis benefit more from exercise therapy than people with longer symptom duration: An individual participant data meta-analysis from the OA trial bank. Osteoarthritis Cartilage. 2024;32(12):1620–27. 10.1016/j.joca.2024.07.007 39032625

[48] Chang AH, Lee JJ, Chmiel JS, Almagor O, Song J, Sharma L. Association of Long-term Strenuous Physical Activity and Extensive Sitting With Incident Radiographic Knee Osteoarthritis. JAMA Netw Open. 2020;3(5):e204049. 10.1001/jamanetworkopen.2020.4049 PMC7199114 32364594

[49] Bitar S, Omorou AY, Van Hoye A, Guillemin F, Rat AC. Five-year Evolution Patterns of Physical Activity and Sedentary Behavior in Patients with Lower-limb Osteoarthritis and Their Sociodemographic and Clinical Correlates. J Rheumatol. 2020;47(12):1807–14. 10.3899/jrheum.190854 32173658

[50] Duong V, Hunter DJ, Nicolson PJA. Predictors and Measures of Adherence to Core Treatments for Osteoarthritis. Clin Geriatr Med. 2022;38(2):345–60. 10.1016/j.cger.2021.11.007 35410684

[51] Cronstrom A, Ingelsrud LH, Nero H, Lohmander LS, Ignjatovic MM, Dahlberg LE, Kiadaliri A. Interpretation threshold values for patient-reported outcomes in patients participating in a digitally delivered first-line treatment program for hip or knee osteoarthritis. Osteoarthr Cartil Open. 2023;5(3):100375. 10.1016/j.ocarto.2023.100375 PMC10238848 37275788

[52] Wright AA, Cook CE, Baxter GD, Dockerty JD, Abbott JH. A comparison of 3 methodological approaches to defining major clinically important improvement of 4 performance measures in patients with hip osteoarthritis. J Orthop Sports Phys Ther. 2011;41(5):319–27. 10.2519/jospt.2011.3515 21335930

[53] Skou ST, Bricca A, Roos EM. The impact of physical activity level on the short- and long-term pain relief from supervised exercise therapy and education: a study of 12,796 Danish patients with knee osteoarthritis. Osteoarthritis Cartilage. 2018;26(11):1474–8. 10.1016/j.joca.2018.07.010 30076884

[54] Liu SH, Driban JB, Eaton CB, McAlindon TE, Harrold LR, Lapane KL. Objectively Measured Physical Activity and Symptoms Change in Knee Osteoarthritis. Am J Med. 2016;129(5):497–e505491. 10.1016/j.amjmed.2015.12.029 PMC6768827 26844633

[55] Liu SH, Eaton CB, Driban JB, McAlindon TE, Lapane KL. Comparison of self-report and objective measures of physical activity in US adults with osteoarthritis. Rheumatol Int. 2016;36(10):1355–64. 10.1007/s00296-016-3537-9 27435920

[56] Kiadaliri A, Englund M. Osteoarthritis and risk of hospitalization for ambulatory care-sensitive conditions: a general population-based cohort study. Rheumatology (Oxford). 2021;60(9):4340–7. 10.1093/rheumatology/keab161 PMC8410004 33590848

[57] Gustafsson K, Kvist J, Eriksson M, Dahlberg LE, Rolfson O. Socioeconomic status of patients in a Swedish national self-management program for osteoarthritis compared with the general population-a descriptive observational study. BMC Musculoskelet Disord. 2020;21(1):10. 10.1186/s12891-019-3016-z PMC6945568 31906904

