# Supplementary material for: Trajectories of physical activity following digital education and exercise therapy—a longitudinal registry study among 15,197 persons with knee or hip osteoarthritis
Source: BMC Musculoskelet Disord. 2026 Oct 11;27:872. doi: 10.1186/s12891-026-10590-w (PMC13670715; doi:10.1186/s12891-026-10590-w)

Table S1. The response options for two physical activity questions and corresponding total physical activity score. Green/red colours indicate reaching/ not reaching the recommended physical activity level.

			Daily physical activity						
	Category		1	2	3	4	5	6	7
		Minute	0	<30	30-60	60-90	90-150	150-300	>300
Exercise	1	0	3	4	5	6	7	8	9
	2	<30	5	6	7	8	9	10	11
	3	30-60	7	8	9	10	11	12	13
	4	60-90	9	10	11	12	13	14	15
	5	90-120	11	12	13	14	15	16	17
	6	>120	13	14	15	16	17	18	19

Table S2. Number (%) of responses to the physical activity (PA) questions per participant.

# of responses per participant	2	3	4	5
All, n=15,197	3,884 (25.6)	3,446 (22.7)	3,389 (22.3)	4,478 (29.5)
Not reaching the recommended PA level at enrolment, n=7,314	1,836 (25.1)	1,736 (23.7)	1,633 (22.3)	2,109 (28.8)
Reaching the recommended PA level at enrolment, n=7,883	2,048 (26.0)	1,710 (21.7)	1,756 (22.3)	2,369 (30.1)

Table S3. Fit statistics for trajectory models.

# of groups	Absolute BIC	Relative entropy	APPA	OCC	% of the sample	Mismatch, %
1	62711	-	-	-	100	-
2	57331	0.722	0.934/0.916	17/9	42.8/57.2	2.0/-2.0
3	56618	0.778	0.948/0.863/0.920	30/11/36	38.8/41.0/20.2	-0.5/-3.6/4.1
4	56417	0.777	0.913/0.833/0.923/0.808	25/33/17/21	30.4/11.9/41.1/16.5	-0.7/1.1/-0.4/0.06
5	56361	0.740	0.947/0.788/0.683/0.830/0.711	33/58/6/16/22	35.7/6.5/33.6/10.5/13.8	-0.1/-0.5/-8.5/12.8/-3.6

BIC: Bayesian information criterion, APPA: Average posterior probability of assignment, OCC: Odds of correct classification.

Bold values indicate the fit statistics that did not meet pre-defined conditions.

[a] Variance matrix was nonsymmetric or highly singular

Table S4. Baseline characteristics of participants assigned to different physical activity trajectories.

	DPA	IPA	PHPA	PLPA
N (%)	1812 (11.9)	2513 (16.5)	6253 (41.2)	4619 (30.4)
Female, n (%)	1390 (76.7)	1923 (76.5)	4551 (72.8)	3573 (77.4)
Age, mean (SD)	64.6 (9.0)	64.9 (8.5)	63.5 (8.8)	65.0 (8.8)
Living in metropolitan cities, n (%)	344 (19.0)	479 (19.1)	1277 (20.4)	829 (18.0)
Education, n (%)				
Less than high school	160 (8.8)	246 (9.8)	396 (6.3)	524 (11.3)
High school	651 (35.9)	928 (36.9)	1992 (31.9)	1930 (41.8)
College/university	1001 (55.2)	1339 (53.3)	3865 (61.8)	2165 (46.9)
Body mass index, n (%)				
<25	681 (37.6)	817 (32.5)	2906 (46.5)	1122 (24.3)
25-29.9	754 (41.6)	1111 (44.2)	2480 (39.7)	1864 (40.4)
≥30	377 (20.8)	585 (23.3)	867 (13.9)	1633 (35.4)
Patient-acceptable symptom state:yes, n (%)	327 (18.1)	457 (18.2)	1116 (17.9)	730 (15.8)
Diabetes, n (%)	92 (5.1)	153 (6.1)	237 (3.8)	362 (7.8)
Lung diseases, n (%)	198 (10.9)	258 (10.3)	557 (8.9)	571 (12.4)
Balance troubles, n (%)	59 (3.3)	84 (3.3)	144 (2.3)	217 (4.7)
Rheumatoid arthritis, n (%)	88 (4.9)	111 (4.4)	245 (3.9)	242 (5.2)
Cardiovascular diseases, n (%)	134 (7.4)	174 (6.9)	420 (6.7)	410 (8.9)
Knee as the index joint, n (%)	1075 (59.3)	1525 (60.7)	3774 (60.4)	2712 (58.7)
Walking difficulties, n (%)	1188 (65.6)	1655 (65.9)	3674 (58.8)	3422 (74.1)
Fear of movement, n (%)	274 (15.1)	371 (14.8)	911 (14.6)	747 (16.2)
Wish for surgery, n (%)	307 (16.9)	355 (14.1)	757 (12.1)	876 (19.0)
Motivation (0-10), mean (SD)	9.3 (1.2)	9.2 (1.3)	9.5 (1.1)	9.0 (1.6)
NRS pain (0-10), mean (SD)	5.1 (2.0)	5.1 (1.9)	4.8 (1.9)	5.4 (1.9)
30s CST, mean (SD)	12.6 (4.1)	12.4 (4.0)	13.7 (4.5)	11.6 (4.0)
KOOS-12/HOOS-12 pain (0-100), mean (SD)	52.8 (16.4)	52.8 (15.4)	55.1 (15.6)	50.0 (16.4)
KOOS-12/HOOS-12 function (0-100), mean (SD)	62.4 (19.0)	62.1 (18.4)	65.8 (17.8)	58.1 (19.0)
KOOS-12/HOOS-12 QoL (0-100), mean (SD)	46.7 (17.0)	46.0 (16.5)	48.5 (16.0)	42.4 (17.1)
Activity impairment (0-10), mean (SD)	3.9 (2.4)	4.0 (2.3)	3.6 (2.3)	4.4 (2.4)
EQ-5D-5L index (-0.31–1), mean (SD)	0.85 (0.17)	0.84 (0.16)	0.87 (0.14)	0.81 (0.19)
Total physical activity score (3-19), mean (SD)	13.1 (2.6)	8.4 (1.7)	14.6 (2.9)	7.4 (2.0)

SD: Standard deviation; DPA: Declining physical activity; IPA: Increasing physical activity; PHPA: Persistently high physical activity; PLPA: Persistently low physical activity, QoL: Quality of life.

Table S5. Mean changes (95% CI) in patient-reported outcomes from enrolment across different physical activity trajectories, adjusted for covariates and the outcome of interest at enrolment.

	DPA	IPA	PHPA	PLPA
**KOOS-12/HOOS-12 pain (0–100)**				
3-month	7.8 (7.0, 8.5)	8.3 (7.7, 8.9)	8.8 (8.4, 9.2)	7.3 (6.8, 7.7)
6-month	9.0 (8.1, 9.9)	10.4 (9.7, 11.2)	10.5 (10.1, 11.0)	8.3 (7.7, 8.8)
9-month	9.4 (8.4, 10.4)	10.8 (10.0, 11.7)	11.0 (10.5, 11.5)	9.2 (8.6, 9.9)
12-month	9.7 (8.4, 11.0)	9.5 (8.4, 10.7)	10.6 (10.0, 11.2)	8.9 (8.1, 9.7)
**KOOS-12/HOOS-12 function (0–100)**				
3-month	6.5 (5.8, 7.3)	6.9 (6.2, 7.5)	7.4 (7.0, 7.8)	6.2 (5.7, 6.7)
6-month	7.1 (6.2, 8.0)	8.0 (7.3, 8.8)	8.3 (7.8, 8.8)	6.6 (6.1, 7.2)
9-month	5.7 (4.6, 6.7)	8.3 (7.4, 9.2)	8.5 (7.9, 9.0)	7.2 (6.5, 7.8)
12-month	6.7 (5.4, 8.0)	6.8 (5.6, 8.1)	7.6 (7.0, 8.2)	6.9 (6.1, 7.7)
**KOOS-12/HOOS-12 QoL (0–100)**				
3-month	4.9 (4.2, 5.6)	6.3 (5.7, 6.9)	6.2 (5.8, 6.6)	5.4 (5.0, 5.8)
6-month	6.3 (5.5, 7.1)	8.4 (7.7, 9.1)	8.0 (7.6, 8.5)	6.6 (6.1, 7.2)
9-month	6.6 (5.6, 7.5)	9.5 (8.6, 10.3)	8.9 (8.4, 9.4)	8.0 (7.3, 8.6)
12-month	6.9 (5.7, 8.2)	8.1 (6.9, 9.2)	9.0 (8.4, 9.7)	7.8 (7.1, 8.6)
**30s chair stand test**				
3-month	4.9 (4.7, 5.2)	5.1 (4.9, 5.3)	5.4 (5.2, 5.5)	4.8 (4.7, 5.0)
6-month	5.9 (5.6, 6.2)	6.4 (6.1, 6.6)	6.5 (6.3, 6.6)	5.9 (5.7, 6.1)
9-month	6.1 (5.8, 6.4)	6.6 (6.3, 6.9)	6.9 (6.7, 7.1)	6.0 (5.8, 6.3)
12-month	6.0 (5.6, 6.4)	6.7 (6.4, 7.1)	7.2 (7.0, 7.4)	6.4 (6.1, 6.6)
**EQ-5D-5L index score (-0.31–1)**				
3-month	0.04 (0.03, 0.04)	0.05 (0.04, 0.05)	0.04 (0.03, 0.04)	0.04 (0.04, 0.05)
6-month	0.04 (0.03, 0.05)	0.05 (0.05, 0.06)	0.04 (0.04, 0.04)	0.05 (0.04, 0.05)
9-month	0.03 (0.02, 0.04)	0.05 (0.05, 0.06)	0.04 (0.04, 0.05)	0.04 (0.04, 0.05)
12-month	0.03 (0.02, 0.05)	0.04 (0.03, 0.05)	0.04 (0.04, 0.05)	0.04 (0.04, 0.05)
**Activity impairment (0–10)**				
3-month	-0.9 (-1.0, -0.8)	-1.0 (-1.1, -0.9)	-1.0 (-1.1, -1.0)	-0.8 (-0.9, -0.8)
6-month	-0.9 (-1.0, -0.8)	-1.2 (-1.3, -1.1)	-1.2 (-1.3, -1.1)	-0.9 (-1.0, -0.9)
9-month	-0.8 (-1.0, -0.7)	-1.3 (-1.4, -1.2)	-1.2 (-1.3, -1.1)	-1.0 (-1.1, -0.9)
12-month	-0.9 (-1.1, -0.7)	-1.1 (-1.3, -0.9)	-1.2 (-1.3, -1.1)	-1.0 (-1.1, -0.9)

DPA: Declining physical activity; IPA: Increasing physical activity; PHPA: Persistently high physical activity; PLPA: Persistently low physical activity, QoL: Quality of life.

Figure S1. Patterns of reaching the recommended physical activity level from enrolment up to one year after enrolment. Yes: reaching the recommended physical activity level; No: Not reaching the recommended physical activity level; MR: missing response.

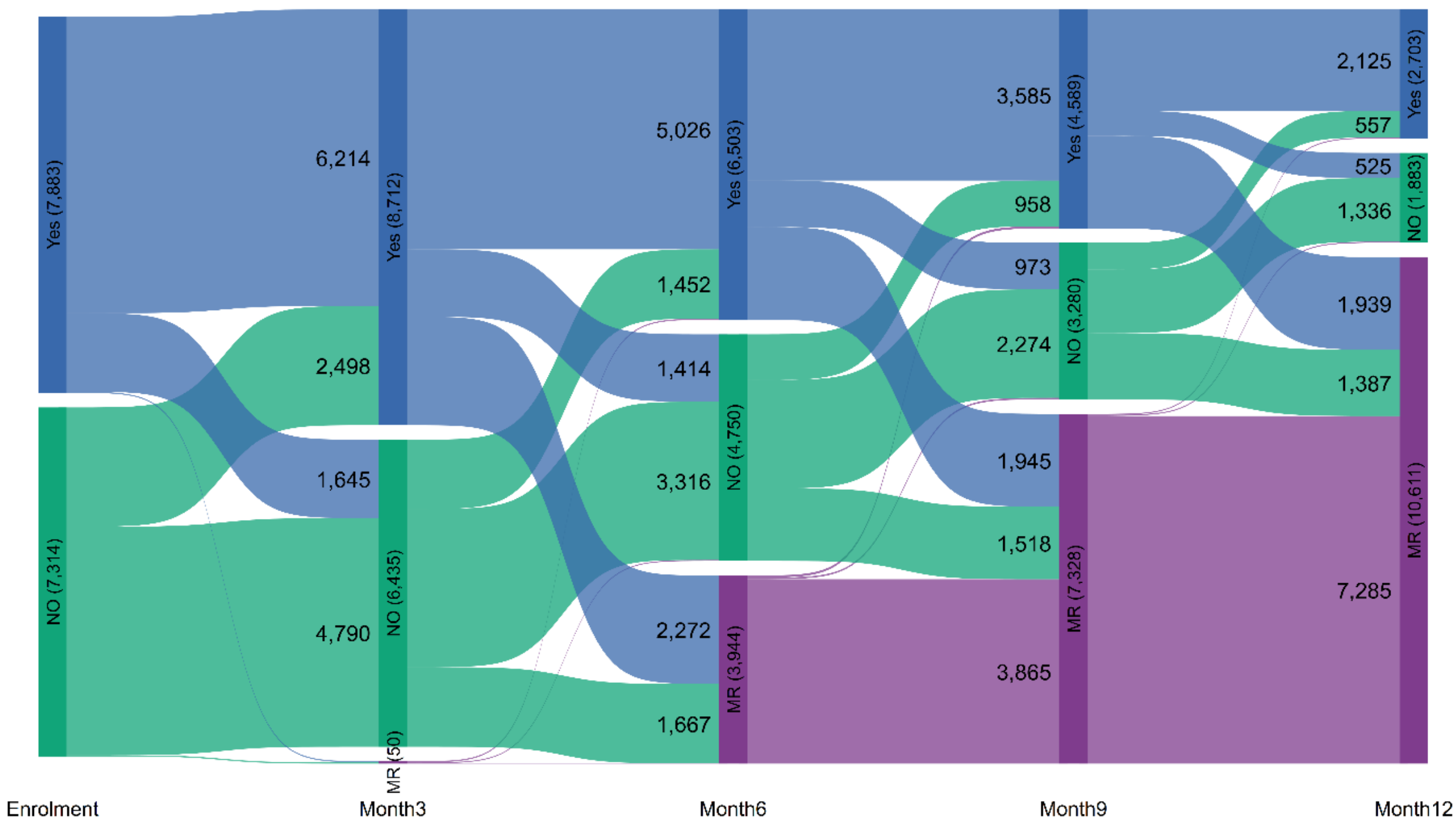

Figure S2. Mean (95% confidence intervals) total physical activity scores across physical activity trajectories. Dashed black line indicates the cut-off value for the recommended physical activity level.

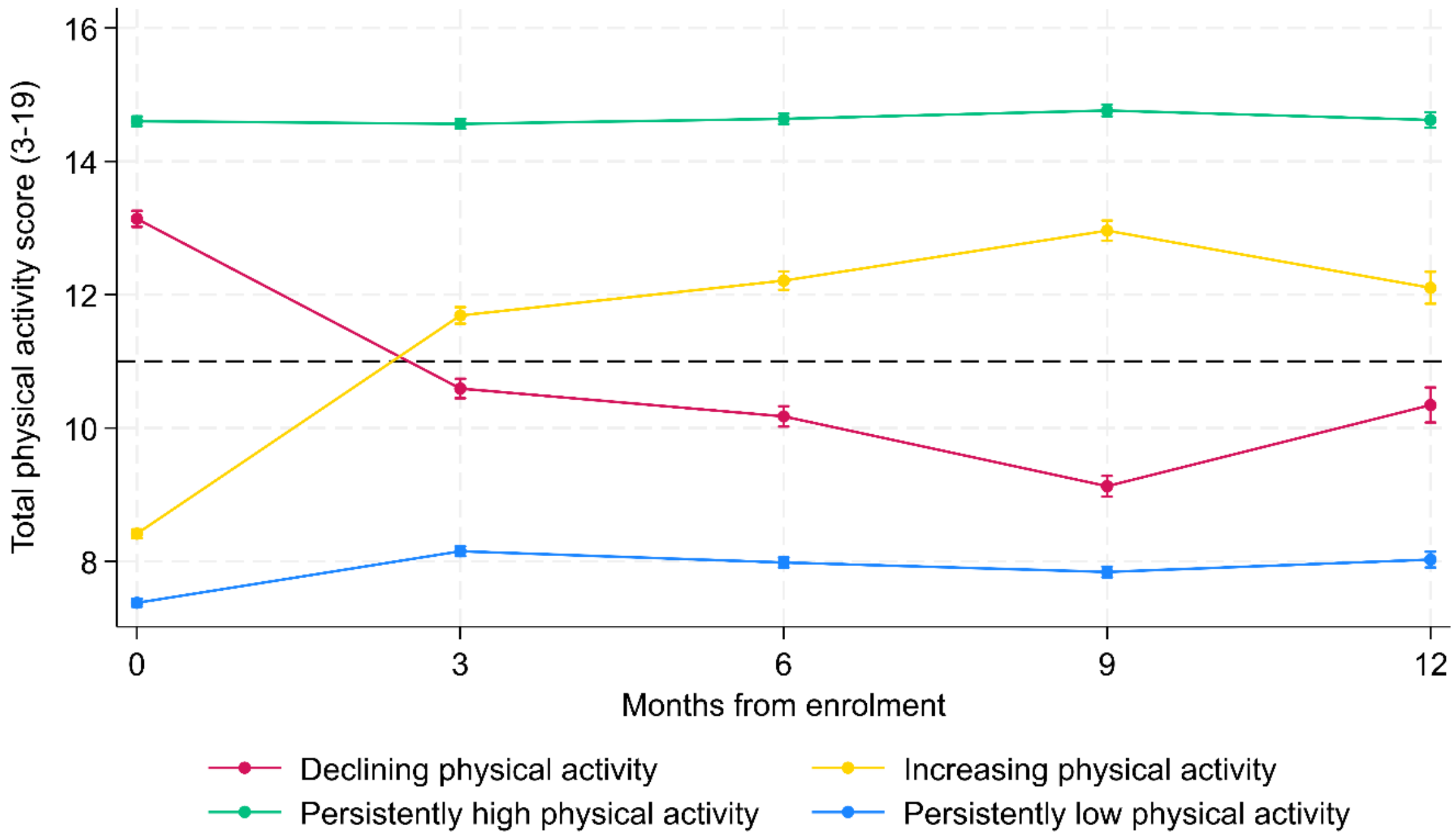

Supplement: Supplementary file 1 [file 12891_2026_10590_MOESM1_ESM.pdf]
